# Improved synthesis of concave cubic gold nanoparticles and their applications for Raman analysis of surfaces

**DOI:** 10.1039/c9ra03012c

**Published:** 2019-06-13

**Authors:** Jan Krajczewski, Mateusz Kędziora, Karol Kołątaj, Andrzej Kudelski

**Affiliations:** Faculty of Chemistry, University of Warsaw ul. Pasteura 1 02-093 Warsaw Poland karolkolataj@gmail.com +48-225526434

## Abstract

In this contribution, we present a modification of the procedure for producing concave cubic gold (cc-Au) nanoparticles; this modification significantly increases the homogeneity of the product obtained. The synthesis of cc-Au is carried out by the slow growth of seed nanostructures in a solution containing chloroauric acid, silver nitrate, ascorbic acid and hexadecyltrimethylammonium chloride. We show that, when nanoparticles synthesized in a solution containing both chloroauric acid and copper chloride (with the molar ratio equal to *ca.* 10 : 1) are used as seeds (instead of seeds formed without the addition of copper), one can observe a significant increase in the homogeneity of the cc-Au nanostructures formed. The resulting cc-Au, and cc-Au@Ag nanoparticles (cc-Au covered by a nanometric layer of silver) as well, have been used as plasmonic cores in nanoresonators dedicated for shell-isolated nanoparticle-enhanced Raman spectroscopy (SHINERS). To our knowledge, the SHINERS nanoresonators produced in this work display a homogeneity that is significantly better than that of any anisotropic SHINERS nanostructures previously synthesized without the subsequent complex process of purifying the nanoparticles. Concave cubic nanoparticles were about 5 times more efficient as electromagnetic nanoresonators than spherical nanostructures of a similar size formed from the same material.

## Abstract 2

In this contribution, we present a modification of the procedure for producing concave cubic gold (cc-Au) nanoparticles; this modification significantly increases the homogeneity of the product obtained.

## Graphical abstract

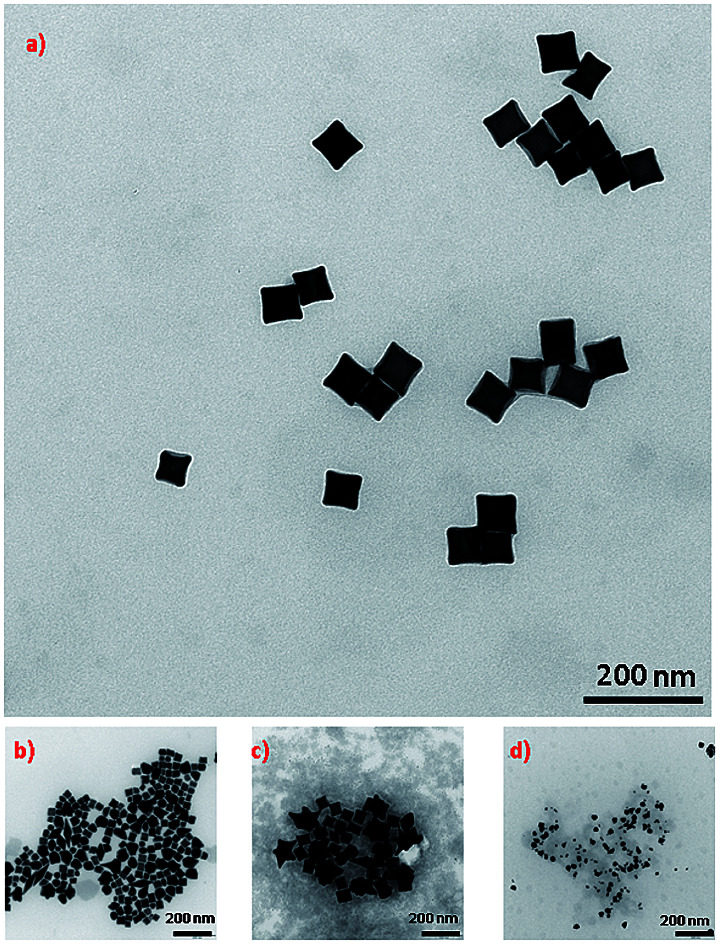

## 1. Introduction

When electromagnetic radiation interacts with nanoparticles formed from metals with a negative real and small positive imaginary dielectric constant (for example, nanoparticles of gold or silver), it induces a collective oscillation of surface conduction electrons, called surface plasmons.^1–4^ The conditions under which effective surface plasmon resonance is observed strongly depend on the material from which the plasmonic structure is formed, and on its shape and size. Since in many cases the wavelength of the radiation used to excite surface plasmon resonance should be within a specific range, for example, within the transparency window of human tissues, different plasmonic nanoparticles of various sizes and shapes are synthesized for different applications. One of the most important functionalities of plasmonic nanostructures caused by the excitation of surface plasmons is a local enhancement of the electromagnetic field in some places in the proximity of the illuminated plasmonic nanoparticles. This field enhancement leads to a significant increase in the efficiency of several optical processes (such as: fluorescence, second harmonic generation, Raman scattering, Raman optical activity, hyper-Raman scattering, coherent anti-Stokes Raman scattering, and infrared absorption) for molecules in close proximity to the plasmonic nanostructures.^1,5–14^

The wavelength of the radiation inducing the surface plasmon resonance in anisotropic nanostructures may be changed across a significantly broader range than for spherical objects. Moreover, the local field enhancement generated by anisotropic plasmonic nanostructures is usually larger than that generated by spherical objects (the highest field enhancement is usually observed at the sharp edges and apexes of anisotropic nanoparticles). Therefore, the synthesis of various anisotropic plasmonic nanostructures is very important from a practical point of view, and such processes have been studied by many groups worldwide. It is worth mentioning that, when plasmonic nanostructures are applied for quantitative analytical measurements, the product obtained must be homogenous and reproducible, and so these issues must be taken into account when developing new synthetic procedures.

In this contribution, we present a modified method for synthesizing concave cubic gold (cc-Au) nanoparticles that yields a raw product (before subsequent complex purification) with significantly higher homogeneity than that obtained using previously applied procedures. The proposed modification consists of using copper-modified seed nanoparticles on which the formation of cc-Au is carried out. TEM analysis confirmed that the cc-Au nanoparticles thus formed display significantly higher homogeneity. The resulting cc-Au nanostructures and cc-Au nanostructures covered with a thin layer of silver were tested as nanoresonators for inducing a local increase in the efficiency of Raman signal generation. We found that nanoparticles having a concave cubic shape are about 5 times more efficient as electromagnetic nanoresonators than analogous spherical nanoparticles of similar size. The nanoparticles obtained were also tested as plasmonic cores in nanoresonators for what is known as shell-isolated nanoparticle-enhanced Raman spectroscopy (SHINERS).^15–18^ In this technique, metallic plasmonic cores are coated by a nanometric, chemically inert protective layer (for example SiO_2_).^15–17^ The deposition of such a layer prevents direct contact between the molecules of the analyte and the metallic cores (such direct contact between the metal and certain molecules, such as DNA or proteins, leads to the denaturation of the DNA or proteins) and prevents any agglomeration of plasmonic nanoparticles. We found that the cc-Au and cc-Au@Ag nanoparticles could be easily and controllably covered by a silica layer, and hence have the potential to be used for synthesizing new, very active and homogeneous nanomaterials for Raman analyses of surfaces.

## 2. Experimental section

### 2.1. Materials

Silver nitrate, hydrochloric acid, ethanol, sodium citrate and citric acid were purchased from POCH S. A. Copper chloride, ascorbic acid, 16-mercaptohexadecanoic acid, dimethylamine, sodium borohydride, hexadecyltrimethylammonium chloride (CTAC), tetraethyl orthosilicate (TEOS), and 4-mercaptobenzoic acid (*p*-MBA) were acquired from Sigma-Aldrich. A 30% HAuCl_4_ solution in dilute HCl (99.99% trace metals basis) and platinum sheets were purchased from the Polish State Mint. The water used in all the experiments was purified in the Millipore Milli-Q manner. All materials were of high purity and were used as received without further purification or treatment.

### 2.2. Synthesis of concave cubic gold nanoparticles

The synthesis of concave cubic gold nanoparticles was carried out by a slow growth of seed nanostructures in a solution containing chloroauric acid, silver nitrate, ascorbic acid and CTAC.^19^ The synthesis of the seed nanoparticles was carried according to the modified method proposed by Zhang *et al.* – with an important difference, that a solution of copper chloride was also added to the reaction mixture (moreover, the addition of salts of other metals was also tested – *vide infra*). Briefly, 0.25 ml of a 10 mM HAuCl_4_, 25 μl of a 10 mM CuCl_2_ and 0.10 ml of a 0.50 M citric acid solution were added to 10 ml of a 50 mM CTAC solution. Then, 0.25 ml of a freshly-prepared ice cold 25 mM NaBH_4_ solution was added under vigorous stirring. Stirring was continued for 2 minutes. Subsequently, the solution was transferred to a thermostat and kept at 70 °C for 2 hours while slowly being stirred. After the reaction, the solution was pale red, which indicates the formation of spherical gold nanoparticles; these were used in the following step as seed nanoparticles. 0.1 ml of the sol of synthesised seed nanoparticles was added to a mixture of: 10 ml of a 0.10 M CTAC solution, 0.1 ml of a 10 mM silver nitrate solution, 0.5 ml of a 10 mM HAuCl_4_ solution, 0.2 ml of a 1 M hydrochloric acid, and 0.1 ml of a 0.10 M ascorbic acid solution. It is worth mentioning that only 0.45% of gold was introduced to the reaction mixture as seed nanoparticles, the rest: 99.55% as a solution of HAuCl_4_ (moreover, whereas in the sol of seed nanoparticles the molar ratio of Cu and Au was equal to 1 : 10, in the final growth solution the molar ratio of Cu and Au was equal to *ca.* 1 : 2200). After introducing the sol of seed nanoparticles, the reaction mixture obtained was manually stirred for 2 seconds. Then, the mixture was transferred to a thermostat and kept at 30 °C for 2 h, without stirring. During this process, the solution changed colour – from practically transparent to an intense blue. The nanoparticles obtained were cleaned from the excess of CTAC by two centrifugations for 7 min at 10^4^*g* and by redispersing the concentrated nanoparticles in water.

### 2.3. Synthesis of spherical gold nanoparticles

Spherical gold nanoparticles were synthesized in a similar way like concave cube gold nanoparticles, however, instead of CTAC solution sodium citrate solution was used. Briefly, 0.25 ml of a 10 mM HAuCl_4_, 25 μl of a 10 mM CuCl_2_ and 0.10 ml of a 0.50 M citric acid solution were added to 10 ml of a 50 mM sodium citrate solution. Then, 0.25 ml of freshly-prepared ice cold 25 mM NaBH_4_ solution was added under vigorous stirring. Stirring was continued for 2 minutes. Subsequently, the solution was transferred to thermostat and kept in 70 °C for 2 hours while slowly being stirred. After the reaction, the solution was pale red, which indicates the formation of spherical gold nanoparticles; these were used in the following step as seed nanoparticles. 0.1 ml of the sol of synthesised seed nanoparticles was added to a mixture of: 10 ml of a 0.10 M sodium citrate solution, 0.1 ml of a 10 mM silver nitrate solution, 0.5 ml of a 10 mM HAuCl_4_ solution, 0.2 ml of a 1 M hydrochloric acid, and 0.1 ml of a 0.10 M ascorbic acid solution. After introducing the sol of seed nanoparticles, the obtained reaction mixture was manually stirred for 2 seconds. Subsequently, the reaction mixture was transferred to the thermostat and kept at 30 °C for 2 h, without stirring.

### 2.4. Deposition of silver layer on concave cubic gold nanoparticles

To deposit a layer of silver on the surface of the cc-Au nanoparticles, to 5 ml of the previously obtained sol of cc-Au nanoparticles, 0.625 ml of a 0.10 M ascorbic acid and 1.25 ml of a 1 mM silver nitrate solution were added. The resulting mixture was vigorously stirred in a magnetic stirrer for 2 min. The solution was then kept at 30 °C for 30 min, without stirring. The core–shell nanoparticles were subsequently centrifuged at 10^4^*g* for 10 min, and redispersed in water.

### 2.5. Deposition of silica layer on concave cubic Au and Au@Ag nanoparticles

To deposit thin silica shells on the concave cubic Au or Au@Ag nanoparticles, we used the method developed by Xue *et al.*^20^ Firstly, an ethanolic solution of 16-mercaptohexadecanoic acid was added to the solution of the previously cleaned Au or Au@Ag concave cubic nanoparticles, to a final concentration of 20 μM. Then, the nanoparticles were centrifuged for 10 min at 10^4^*g* and redispersed in a 0.2 mM TEOS solution. An aqueous solution of dimethylamine was added to this solution under constant stirring, to a final dimethylamine concentration of 0.6 M. After 3 h, the concave cubic Au@SiO_2_ or Au@Ag@SiO_2_ nanoparticles thus formed were centrifuged for 10 min at 5000*g* and redispersed in water.

### 2.6. Formation of thiolate monolayers on the platinum surfaces

In measurements carried out to compare the efficiency of various plasmonic structures in enhancing the intensity of the Raman spectra, monolayers formed from 4-mercaptobenzoic acid (*p*-MBA) on platinum surfaces were used as model SERS substrates. To form such monolayers, flame-annealed platinum substrates were immersed in a saturated aqueous solution of *p*-MBA for 1 day. Then, the *p*-MBA-modified platinum surfaces were rinsed with water and allowed to dry in air.

### 2.7. Experimental techniques

Transmission electron microscopy (TEM) analyses of the nanoparticles formed were carried out using a Zeiss LIBRA 120 electron microscope equipped with an in-column OMEGA filter, working at an accelerating voltage of 120 kV. To prepare samples for the TEM measurements, the nanoparticle solutions were deposited onto 300-mesh copper grids coated with a Formvar layer, and the solvent of the deposited solutions was allowed to dry.

Scanning electron microscopy (SEM) analyses of the nanoparticles formed were carried out using a Merlin (Zeiss, Germany) field emission scanning electron microscope (SEM) equipped with an energy-dispersive X-ray microanalysis (EDS) probe (Bruker). The sols of the nanoparticles analysed were deposited on the surface of a graphite substrate, and the solvent was allowed to dry.

Raman spectra were measured with a Horiba Jobin-Yvon Labram HR800 spectrometer coupled with an confocal Olympus BX40 microscope with a long distance 50× objective. The Raman spectrometer was equipped with: a Peltier-cooled CCD detector (1024 × 256 pixels), a 600 groove per mm holographic grating, while a He–Ne laser provided the excitation radiation, at a wavelength of 633 nm.

UV-vis extinction spectra were recorded using a Shimadzu UV-2401PC spectrophotometer.

## 3. Results and discussion

### 3.1. Synthesis of concave cubic gold nanoparticles

**Fig. 1:**
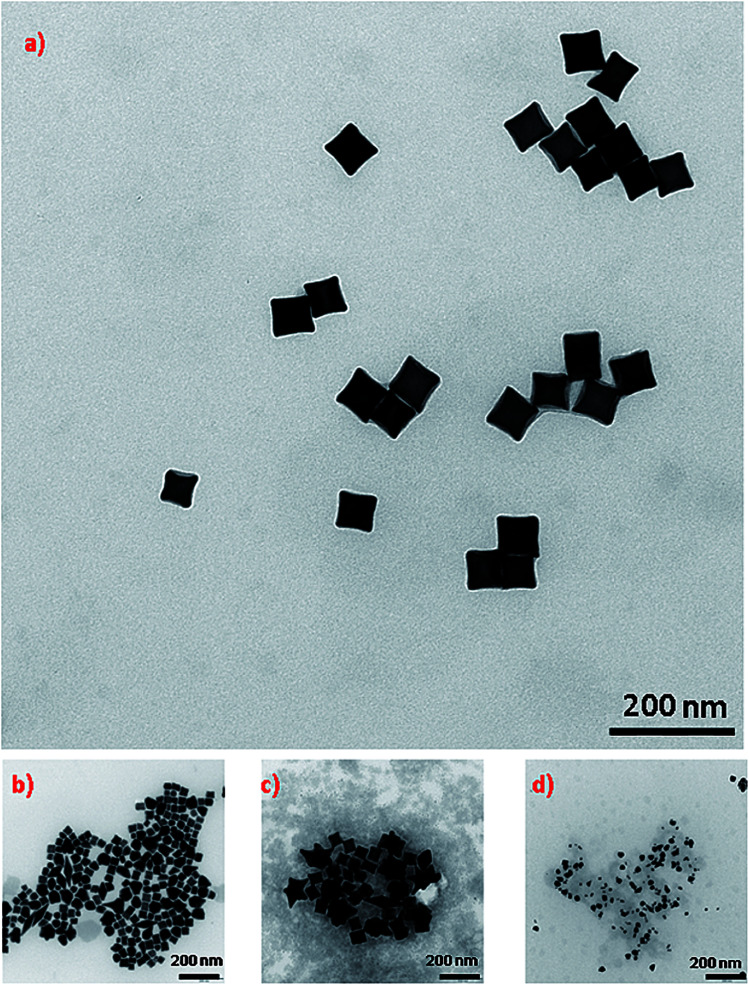
TEM micrographs showing nanoparticles growth in a solution containing HAuCl_4_, AgNO_3_, ascorbic acid and CTAC using: (a) seeds obtained by the reduction of a mixture of solutions of CuCl_2_ and HAuCl_4_ with a molar ratio 1 : 10, (b) seeds obtained by the reduction of only HAuCl_4_ solution, (c) seeds obtained by the reduction of a mixture of solutions of CuCl_2_ and HAuCl_4_ with a molar ratio 1 : 5. (d) TEM micrographs showing nanoparticles growth in a solution containing HAuCl_4_, AgNO_3_, CuCl_2_, ascorbic acid and CTAC using seeds obtained by the reduction of only HAuCl_4_ solution.

As mentioned in the Experimental section, concave cubic gold (cc-Au) nanoparticles were synthesized *via* the seed-mediated growth method (seed nanostructures were introduced to the solution containing chloroauric acid, silver nitrate, ascorbic acid and CTAC – for details, see the Experimental section; the just-obtained mixture was stirred for a short time, and after that was kept at 30 °C for 1 h, without stirring). In the method originally developed by Zhang *et al.* for synthesising cc-Au nanoparticles, single-element gold nanoparticles synthesised by the reduction of HAuCl_4_ by NaBH_4_ in the presence of CTAC were used as seed nanoparticles.^19^ On the basis of an analysis of the TEM images of the product finally formed, we found that, when a small amount of copper salt is added to the solution from which the seed nanoparticles are formed, the final product contains a significantly larger percentage of cc-Au nanoparticles than when single-element gold seed nanoparticles are used (see Fig. 1). For example, the addition of a solution of copper chloride to the solution of chloroauric acid from which the seed nanoparticles are formed, in such an amount that the molar ratio of CuCl_2_ and HAuCl_4_ is equal to 1 : 10 (which seems to be the optimum value), increases the yield of the cc-Au nanoparticles from about 69% to more than 96% (both reaction yields were calculated by analysing the shapes of more than 500 randomly chosen nanoparticles). A further increase of the copper/gold molar ratio (for example, to 1/5) leads to a decrease in the yield of the formation of cc-Au nanoparticles – more dipyramids and desert rose-like objects (aggregates of the expected nanoparticles) are formed (see Fig. 1c). For example, for a Cu/Au molar ratio equal to 1/5, the percentage of cc-Au nanoparticles in the final product is equal to about 65%. EDS measurements show that the ratio of the atomic concentrations of gold and silver in the nanoparticles obtained is equal to 70 : 1, whereas in the reaction mixture that ratio is 5 : 1. This means that the nanoparticles obtained are indeed practically only of gold, and only slightly contaminated with silver.

**Fig. 2:**
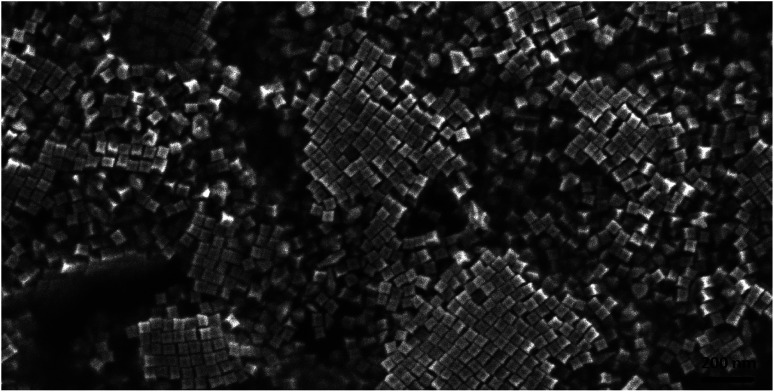
SEM image of a layer of concave cubic nanoparticles deposited on a platinum plate.

An SEM image showing many hundreds of cc-Au nanoparticles formed from seeds obtained from the solution containing the optimal Cu/Au molar ratio (1/10) is shown in Fig. 2. It can be seen that the homogeneity and monodispersity of the nanoparticles obtained, which have not been purified, is very good. We also investigated the case in which copper salt was not added to the solution from which the seed nanoparticles were formed, but where CuCl_2_ was added during the growth stage. In this case, however, the addition of CuCl_2_ induced a decrease in the yield of the formation of cc-Au nanoparticles – practically no concave cubes were formed, with the main product being significantly smaller spherical-like nanoparticles (see Fig. 2d). Although the mechanism of the influence of copper is, as of now, unclear, we believe that it is due to a change in the relative amount of the various facets enclosing the seed nanoparticle (for example, deposited copper can stabilise some facets or can spot growth of some facets). We also studied the impact of the addition of other salts containing 2+ metal ions (such as NiCl_2_, PtCl_2_ and CoCl_2_) to the HAuCl_4_ solution from which the seed nanoparticles are formed on the efficiency of the formation thereon of the cc-Au nanoparticles. Only in the case of the addition of CuCl_2_ we have achieve an increase in the yield of cc-Au nanoparticles.

We also examined the influence of growth time and the temperature at which the growth process is carried out on the yield of cc-Au nanoparticles. We found that an increase in temperature above 30 degrees led to a significant decrease in the reaction yield. We also found that the growth process is already completed after 1 h, so there is no need to carry out this reaction “overnight” as suggested by Zhang *et al.* in their original recipe.^19^

**Fig. 3:**
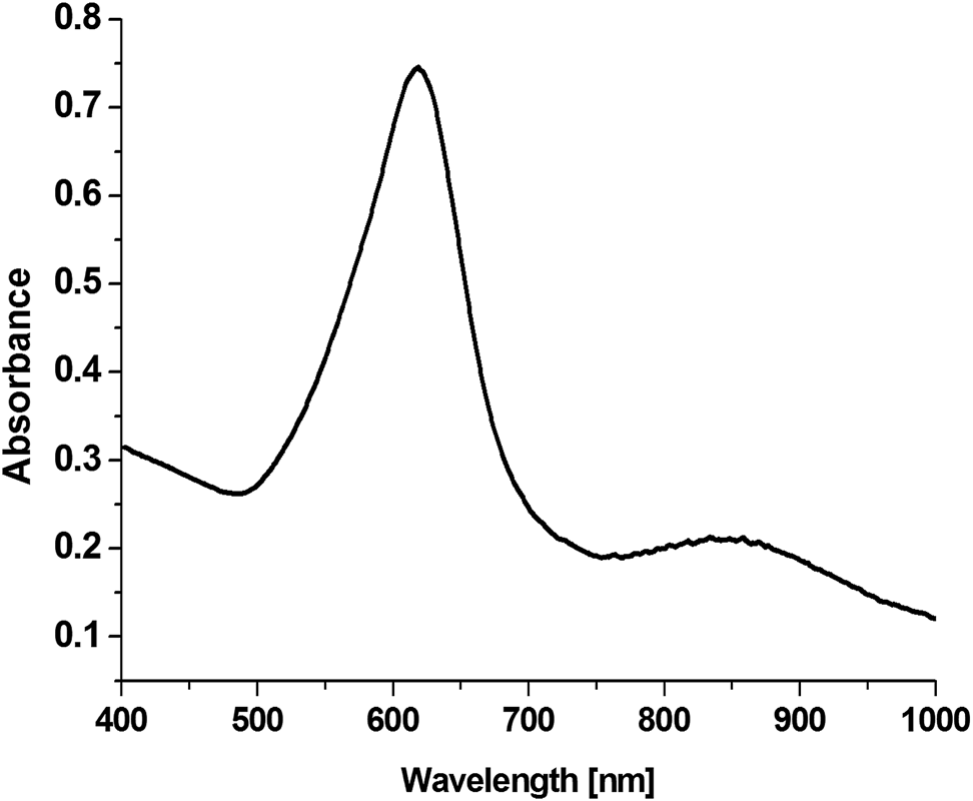
Extinction spectra of a sol of cc-Au nanoparticles having an average edge length of *ca.* 50 nm.

On the basis of an analysis of TEM and SEM images of the nanoparticles formed, one can calculate that the average edge length of the synthetized cc-Au nanoparticles is equal to *ca.* 50 nm (see Fig. 2). The extinction spectra of the sol of cc-Au nanoparticles obtained are shown in Fig. 3. As can be seen, the position of the extinction band for this sol is equal to 616 nm, which is in very good agreement with the position reported by Zhang *et al.* for a sol of cc-Au nanoparticles of similar size.^19^ It is worth mentioning that, as Zhang *et al.* have already shown, the plasmon resonance for cc-Au nanoparticles is red-shifted by 80 nm compared to cubes with flat surfaces and of similar dimensions.^19,21^

### 3.2. Deposition of a silver layer on cc-Au nanoparticles

**Fig. 4:**
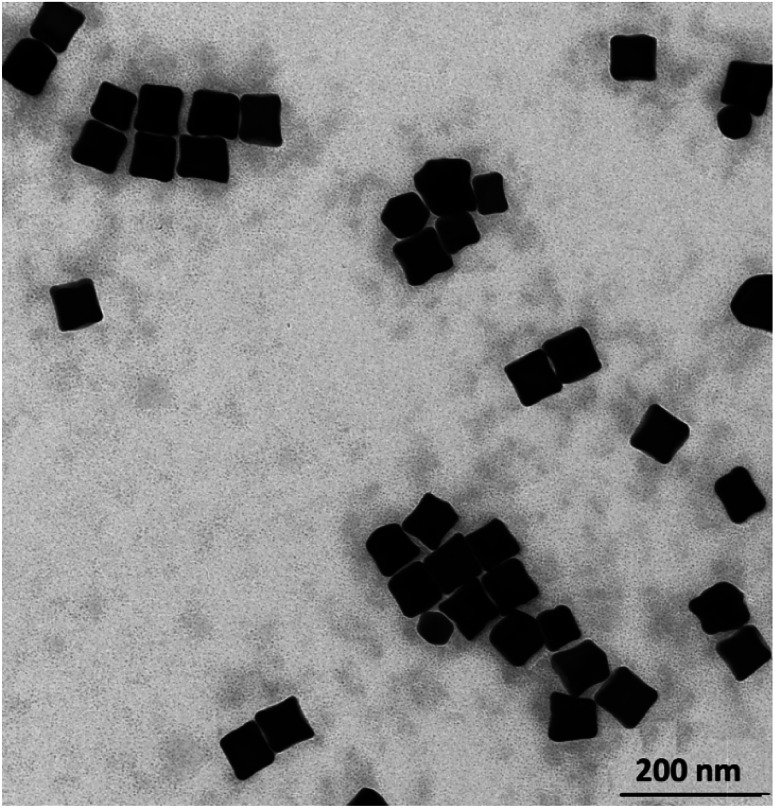
TEM micrograph of concave cubic Au@Ag nanoparticles.

**Fig. 5:**
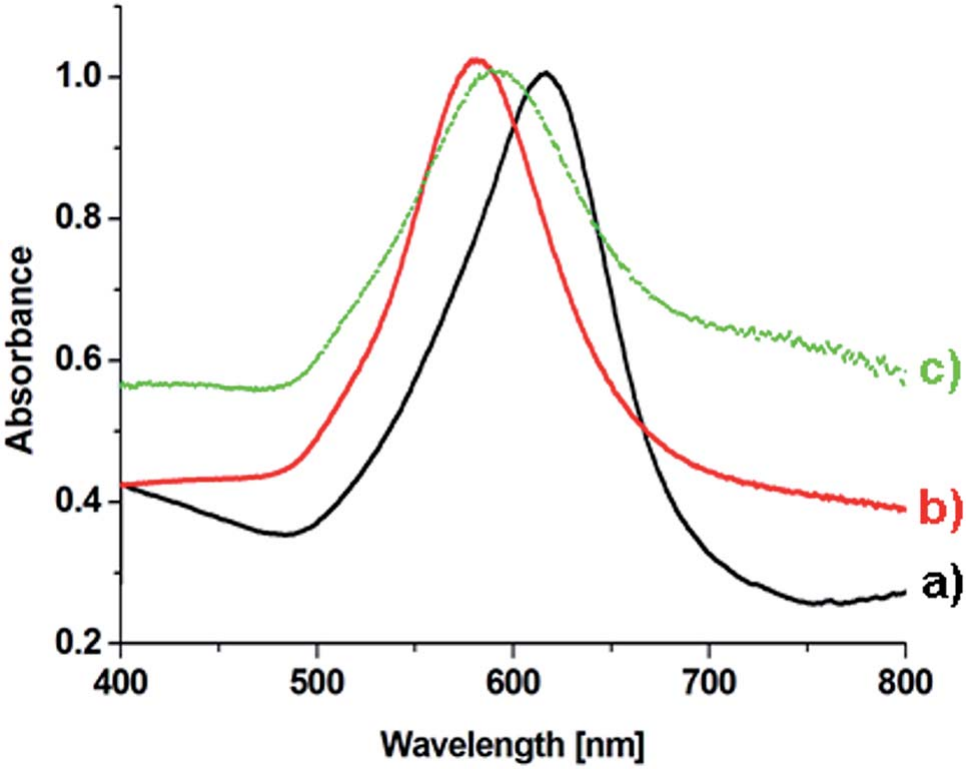
UV-vis extinction spectra of sols containing the following nanoparticles: (a) cc-Au, (b) cc-Au@Ag, and (c) cc-Au@Ag@SiO_2_.

In some cases, the plasmonic nanoparticles were covered with a layer of another plasmonic metal (especially silver, nanoparticles of which support surface plasmon resonance across the whole range of visible radiation) in order to tune their plasmonic properties and to increase the generated local enhancement of the intensity of the electric field. Therefore, we decided to verify whether the cc-Au nanoparticles formed are stable enough to be easily covered with a thin layer of silver. To deposit such a layer, we used the ascorbic acid reduction method. In this method, silver nitrate is slowly reduced in the presence of gold nanoparticles, which are used as seeds for silver growth. Without the presence of the seeds, ascorbic acid is not able to effectively reduce the Ag^+^ ions in these conditions, and so the application of a mild reduction agent, such as ascorbic acid, should practically eliminate the formation of single-element silver nanoparticles. TEM analysis revealed that, in the product obtained after Ag deposition, practically only cubic nanoparticles were present (for the majority of the analysed nanoparticles, it was also well visible in the TEM images that their walls are concave – see Fig. 4) and that the average edge length of the nanoparticles after this process was larger than before the deposition of silver (after carrying out the Ag deposition procedure described in the Experimental section, we observed an increase in the average edge length of the cubic structures of about 4 nm). We found that the change in the concentration of silver nitrate in the reaction mixture affected the value of the increase in the average edge length of the nanoparticles during the reduction process. All of these observations strongly suggest that the core–shell Au@Ag bimetallic nanoparticles are actually formed during this process. The deposition of a silver layer causes a change in the UV-vis extinction spectrum (see Fig. 5) – the maximum of the extinction band is shifted to shorter wavelengths.

### 3.3. Comparison of the efficiency of enhancing of the Raman spectra by spherical and concave cubic nanoparticles; impact of the deposited silver layer

As mentioned in the Introduction, because a very high electric field enhancement is usually observed on the sharp edges and apexes of anisotropic plasmonic nanoparticles, the average enhancement of the Raman signal in the measured SERS spectra (which is locally proportional to the fourth power of the field enhancement) generated by the anisotropic plasmonic nanostructures is usually larger than that generated by spherical plasmonic objects. Therefore, we decided to compare the efficiency of the enhancement of the Raman signal of monolayers of 4-mercaptobenzoic acid (*p*-MBA) formed on a platinum surface generated by a deposited layer of concave and spherical plasmonic nanoparticles synthesised from the same amount of the plasmonic metal and having a similar size (*ca.* 50 nm).

The reasons for choosing monolayers of *p*-MBA on platinum for comparing the SERS activity of various plasmonic nanostructures were: (i) the high stability of such systems – it is well known that thiols are strongly chemisorbed on a platinum surface due to the formation of a metal-sulphur bond and that, even after lengthy immersion in water, only a small part of the *p*-MBA is desorbed from the metal surface, (ii) the high reproducibility of the monolayers obtained, and (iii) platinum, in contrast to many other metals on which *p*-MBA forms regular, stable monolayers (like Ag, Au, Cu), does not effectively support surface plasmon resonance.

**Fig. 6:**
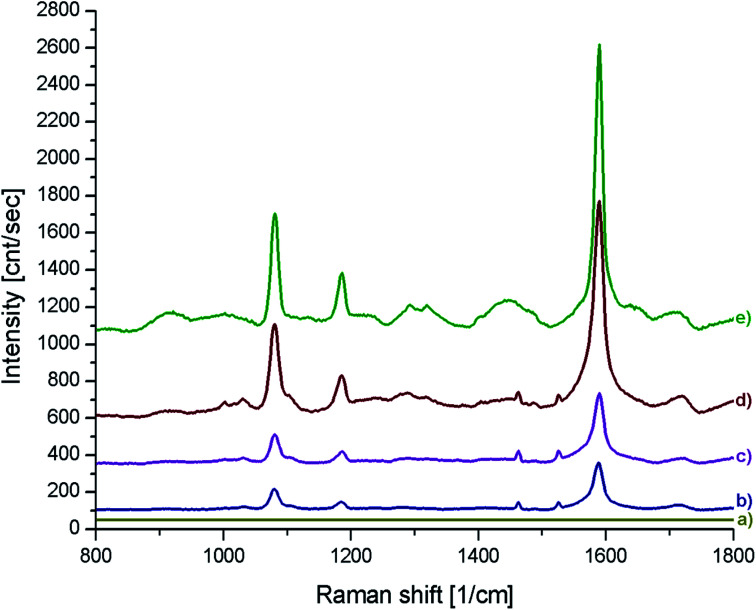
(a) Raman spectrum of a *p*-MBA monolayer on a platinum plate. (b–e) Raman spectra of *p*-MBA monolayers on a platinum plate and covered with: (b) spherical gold nanoparticles, (c) spherical Au@Ag nanoparticles, (d) cc-Au nanoparticles and (e) cc-Au@Ag nanoparticles.

Fig. 6 shows SERS spectra of the *p*-MBA monolayers on platinum covered with concave cubic and spherical Au and Au@Ag nanoparticles having an average size of about 50 nm. The spectra presented in Fig. 6 are the results of averaging 70 spectra measured at various places of the sample. As can be seen in Fig. 6, the obtained SERS spectra are dominated by two intensive bands located at 1070 and 1590 cm^−1^. These bands are assigned to the *ν*_12_ and *ν*_8a_ vibrations of the aromatic ring of *p*-MBA, while the less intensive band at 1185 cm^−1^ is due to the *δ*(C–H) vibrations.^22,23^ For the quantitative determination of the Raman efficiency of various nanoparticles, we calculated the Raman enhancement factor (REF) generated by the deposited plasmonic nanoparticles. Since the deposited nanoresonators geometrically shield the places, where the Raman signal is generated, and only a part of the analysed surface is in contact with the electromagnetic hot spots of the deposited nanoresonators, the actual SERS enhancement factor is significantly larger than the experimentally determined REF generated by the deposited plasmonic nanostructures. The determination of REF was based on the comparison of the intensity of the *p*-MBA band at 1590 cm^−1^ measured with and without deposited plasmonic nanoparticles. As can be seen in Fig. 6, despite the deposition of concave cubic and spherical Au nanoresonators produced from the same number of moles of gold on a very similar area of the *p*-MBA monolayer on platinum, the spectrum (actually the averaged result from 70 measurements) obtained using concave cubic nanoparticles is much more intensive than using spherical nanostructures. REF enhancement factor for spherical gold nanoparticles was estimated to be 6.2 × 10^2^, while for concave gold cubic nanostructures it was almost 5 times higher (2.8 × 10^3^). This means that concave cubic nanoparticles are significantly better nanoresonators for enhancing the intensity of the measured SERS spectra than standard spherical nanoparticles. Despite the deposition of even a very thin silver layer on the gold nanoparticles, changing of the position of their plasmon band in the extinction spectrum is observable (see Fig. 5). As can be seen in Fig. 6, the deposition of a thin silver layer on both concave cubic and semispherical Au nanoparticles only very slightly affected the SERS activity of these nanoparticles. In case of spherical nanoparticles deposition of thin silver shell increased the REF enhancement factor by 56% (from 6.2 × 10^2^ to 9.7 × 10^2^), while in case of cubic nanoparticles the increase in REF is estimated as 35% (from 2.8 × 10^3^ to 3.8 × 10^3^).

### 3.4. Synthesis of concave cubic Au@Ag@SiO_2_ and Au@Ag@SiO_2_ nanoparticles and their application in SHINERS spectroscopy

As mentioned above, one of the important fields of application of plasmonic nanoparticles is their application as nanoresonators for a local increase in the efficiency of certain optical processes, especially Raman scattering. In many cases, there should be no direct contact between the plasmonic metal and the studied molecules, because such interactions lead to significant changes in the objects under analysis (for example, direct interaction of some types of molecules, such as DNA or proteins, with the metal surface leads to their denaturation). Therefore, in some cases, the plasmonic cores are covered by protective layers that eliminate direct contact between the molecules of the analyte and the metallic plasmonic cores, and prevent their undergoing agglomeration. As mentioned in the Introduction, such core–shell structures are used as nanoresonators for shell-isolated nanoparticle-enhanced Raman spectroscopy (SHINERS) measurements.

**Fig. 7:**
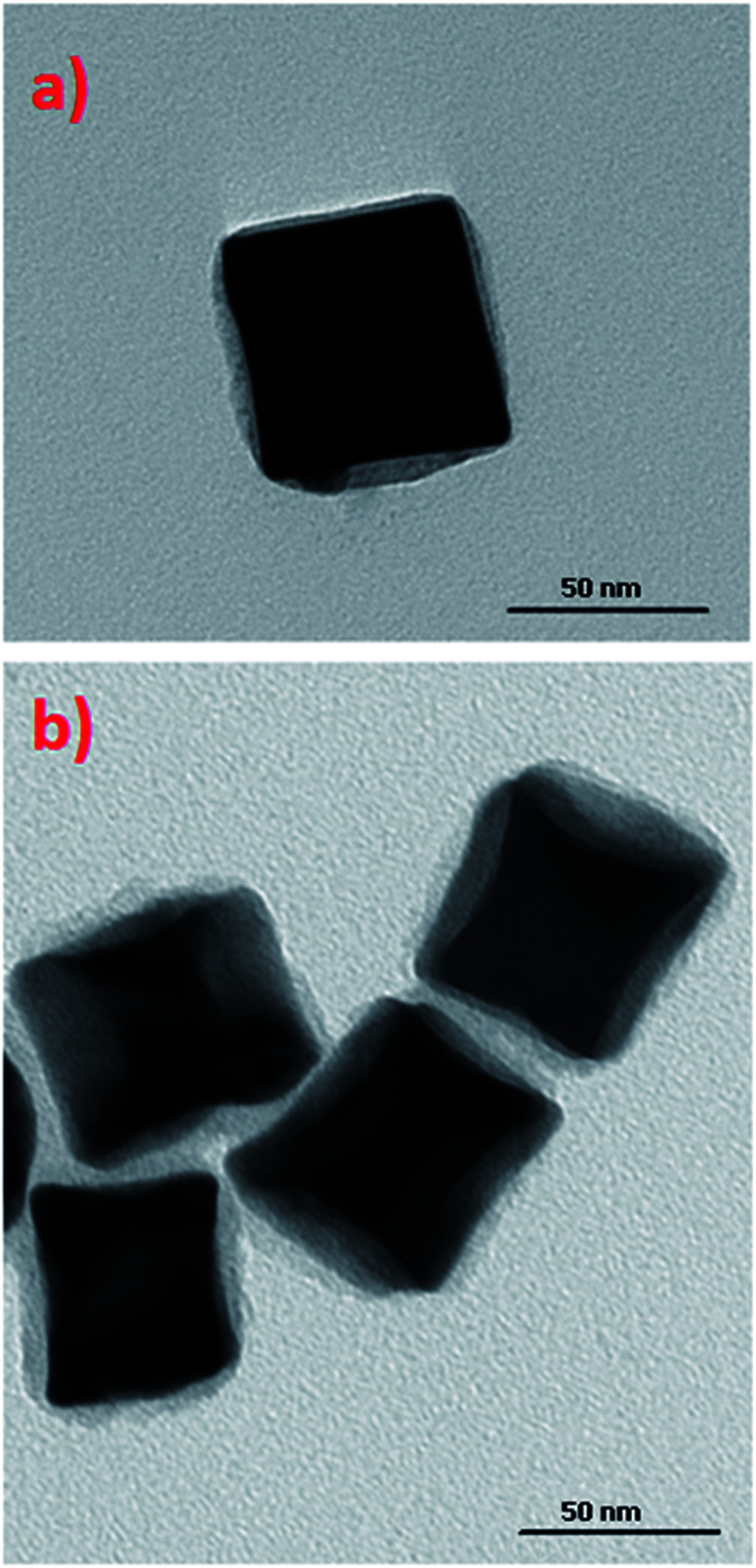
TEM micrographs of obtained: (a) Au@SiO_2_ and (b) Au@Ag@SiO_2_ concave cubic nanoparticles.

To verify the usefulness of cc-Au and cc-Au@Ag nanoparticles as plasmonic cores for SHINERS nanoresonators, we decided to cover them with a very thin SiO_2_ layer using one of the standard methods of SiO_2_ deposition, namely, the decomposition of tetraethyl orthosilicate catalysed by dimethylamine (for details, see the Experimental section – see Fig. 7). We found that the deposition of an SiO_2_ layer using this method does not significantly affect the shape or size of the covered nanoparticles (both cc-Au and cc-Au@Ag nanostructures). This means that cc-Au and cc-Au@Ag nanoparticles can be used as very promising plasmonic cores for the synthesis of SHINERS nanoresonators: they are significantly more SERS active than their analogous spherical nanoparticles, are stable during the covering procedure, and can be easily obtained as monodispersive samples without the need to use complex purification procedures.

The deposition of various dielectric layers on the plasmonic nanoparticles changes the permittivity of the surrounding media, and thus affects the conditions under which surface plasmon resonance is observed. The refractive index of SiO_2_ is higher than that of water; therefore, from a simple model of the plasmon resonance in metal nanoparticles, it could be expected that the deposition of a silica layer should induce a red-shift in the plasmon bands for metal nanoparticles.^24,25^ Our experiments show that, indeed, such a shift of the position of the plasmonic band is observed. However, because the deposited layer is very thin, the shift is very small – only a few nm (for a comparison of the UV-vis extinction spectra of cc-Au@Ag and cc-Au@Ag@SiO_2_ nanoparticles, see Fig. 5).

**Fig. 8:**
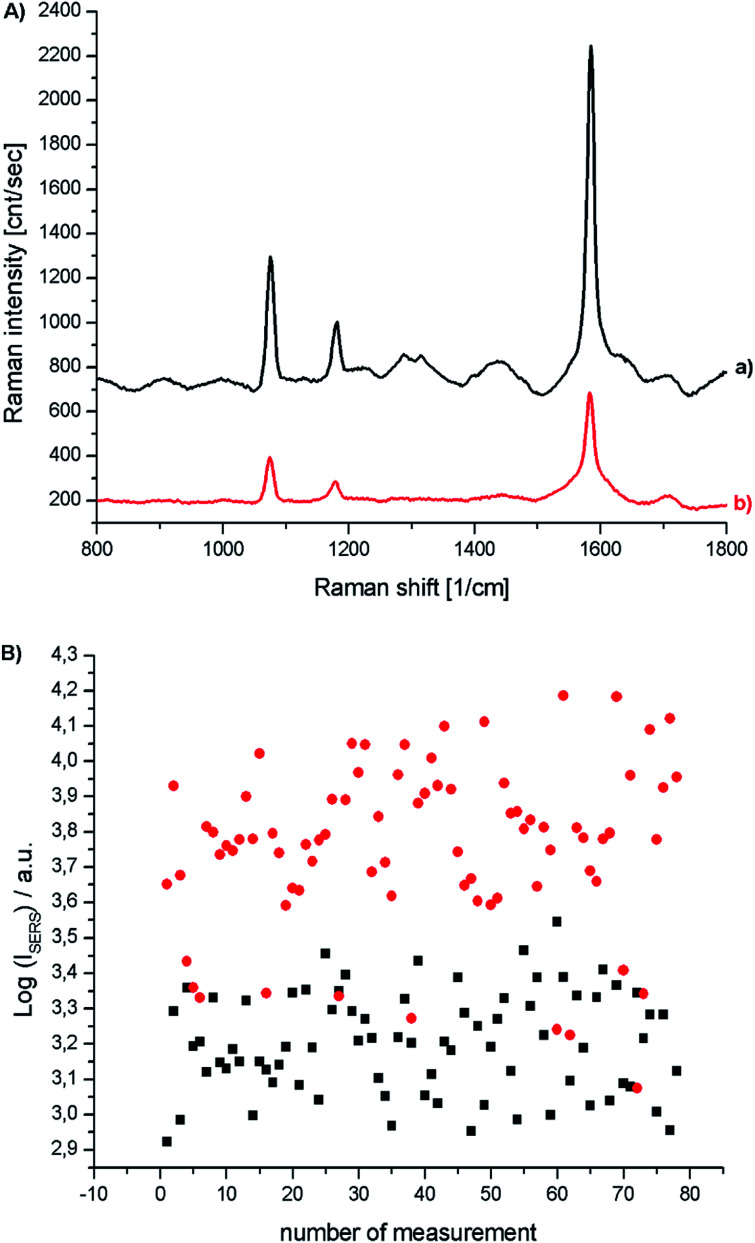
(A) Raman spectra of *p*-MBA monolayers on platinum covered with concave cubic Au@Ag nanoparticles, (a) before and (b) after deposition of a silica layer. (B) Logarithm of the intensity of the strongest *p*-MBA band at 1587 cm^−1^ recorded in each of the series of 78 measurements for a *p*-MBA monolayer on Pt covered with Au@Ag concave cubes nanoparticles (●), and Au@Ag concave cubes nanoparticles after deposition on them of a thin silica layer (■).

The distance between a molecule of the analyte and the surface of the plasmonic nanoresonator is the critical factor in the analytical techniques based on plasmonic nanoparticles such as SERS or MEF (metal enhanced fluorescence). In SERS spectroscopy, the enhancement of the Raman signal decreases with distance as a function of 1/*r*^6^.^26^ Therefore, the deposited layer of silica should be very thin. TEM analysis showed that, using the above-described method of SiO_2_ deposition, we were able to deposit a very thin (below 5 nm) silica layer on the surface of Au@Ag concave cube nanoparticles. In order to determine the impact of the deposited silica layer on the efficiency of the Au@Ag concave cube nanoparticles as plasmonic nanoresonators, we recorded SERS spectra of the *p*-MBA monolayers formed on platinum, on which bare and SiO_2_-covered Au@Ag concave cube nanoparticles had been deposited. An analysis of the spectra recorded showed that the presence of a silica layer causes a decrease in the intensity of the measured Raman spectra, in a range of 50–60%. The irreproducibility of the individual SHINERS measurements for the *p*-MBA monolayers covered with Au@Ag and Au@Ag@SiO_2_ concave cube nanoparticles is presented in Fig. 8B. For better clarity, all of the intensities are presented in a logarithmic scale. As can be seen, the irreproducibility of the intensity of the measured spectrum is significantly smaller than the difference in the SERS activity of the Au@Ag concave cube nanoparticles before and after deposition of the nanometric layer of silica.

## 4. Conclusions

In this work, we present a method for significantly improving the synthesis of gold concave cubic nanoparticles (improving both the yield of the reaction and the homogeneity of the product). This significant increase in the efficiency of the process was achieved by the addition of a certain amount of CuCl_2_ during the process of the formation of seed nanoparticles. The nanoparticles thus obtained were tested as nanoresonators in SERS measurements. We found that gold concave cube nanoparticles are 5 times more active in SERS measurements than standard spherical gold nanostructures of similar size. A further increase in the SERS activity of the structures obtained can be achieved by the deposition of a thin layer of silver on the surface thereof. The structures obtained are stable during the standard process of depositing a silver layer on their surface, and are therefore very promising plasmonic cores for SHINERS nanoresonators used for the Raman analysis of various surfaces.

## Conflicts of interest

There are no conflicts to declare.

## Supplementary Material

## References

[cit1] Etchegoin P. G. and Le Ru E. C., Basic electromagnetic theory of SERS, in Surface enhanced Raman spectroscopy: analytical, biophysical and life science Applications, ed. S. Schlücker, Wiley-VCH, New York, 2011

[cit2] Aroca R. , Surface-Enhanced Vibrational Spectroscopy, John Wiley & Sons Ltd., Chichester, 2006

[cit3] Kottmann J. P., Martin O. J. F., Smith D. R., Schultz S. (2001). Dramatic localized electromagnetic enhancement in plasmon resonant nanowires. Chem. Phys. Lett..

[cit4] Taguchi A., Hayazawa N., Furusawa K., Ishitobi H., Kawata S. (2009). Deep-UV tip-enhanced Raman scattering. J. Raman Spectrosc..

[cit5] Song J. H., Atay T., Shi S., Urabe H., Nurmikko A. V. (2005). Large enhancement of fluorescence efficiency from CdSe/ZnS quantum dots induced by resonant coupling to spatially controlled surface plasmons. Nano Lett..

[cit6] Brolo A. G., Germain P., Hager G. (2002). Investigation of the Adsorption of L-Cysteine on a Polycrystalline Silver Electrode by Surface-Enhanced Raman Scattering (SERS) and Surface-Enhanced Second Harmonic Generation (SESHG). J. Phys. Chem. B.

[cit7] Stranik O., McEvoy H. M., McDonagh C., MacCraith B. D. (2005). Plasmonic enhancement of fluorescence for sensor applications. Sens. Actuators, B.

[cit8] Imae T., Torii H. (2002). In Situ Investigation of Molecular Adsorption on Au Surface by Surface-Enhanced Infrared Absorption Spectroscopy. J. Phys. Chem. B.

[cit9] Addison C. J., Konorov S. O., Brolo A. G., Blades M. W., Turner R. F. B. (2009). Tuning gold nanoparticle self-assembly for optimum coherent anti-stokes Raman scattering and second harmonic generation response. J. Phys. Chem. C.

[cit10] Kim Y. D., Park B., Jung E. C., Jung C. S., Kim M. S. (2005). Charge-transfer effect on surface-enhanced second-harmonic generation. J. Phys. Chem..

[cit11] Leng W., Kelley A. M. (2006). Surface-enhanced hyper-Raman spectra and enhancement factors for three SERS chromophores. SEHRS spectra on Ag films at pulse energies below 2 pJ. J. Am. Chem. Soc..

[cit12] Milojevich C. B., Silverstein D. W., Jensen L., Camden J. P. (2013). Surface-enhanced hyper-Raman scattering elucidates the two-photon absorption spectrum of rhodamine 6G. J. Phys. Chem. C.

[cit13] Osińska K., Pecul M., Kudelski A. (2010). Circularly polarized component in surface-enhanced Raman spectra. Chem. Phys. Lett..

[cit14] Kneipp H., Kneipp J., Kneipp K. (2006). Surface-enhanced Raman optical activity on adenine in silver colloidal solution. Anal. Chem..

[cit15] Li J. F. and Tian Z. Q., Shell-Isolated Nanoparticle-Enhanced Raman Spectroscopy (SHINERS), Front. Surface-Enhanced Raman Scatt. Single Nanoparticles Single Cells, Chichester, 2014, pp. 163–192

[cit16] Koła̧taj K., Krajczewski J., Kudelski A. (2017). Silver Nanoparticles with Many Sharp Apexes and Edges as Efficient Nanoresonators for Shell-Isolated Nanoparticle-Enhanced Raman Spectroscopy. J. Phys. Chem. C.

[cit17] Koła̧taj K., Krajczewski J., Kudelski A. (2018). Dipyramidal-Au@SiO_2_ nanostructures: new efficient electromagnetic nanoresonators for Raman spectroscopy analysis of surfaces. Appl. Surf. Sci..

[cit18] Abdulrahman H. B., Kołątaj K., Lenczewski P., Krajczewski J., Kudelski A. (2016). MnO_2_ - protected silver nanoparticles: new electromagnetic nanoresonators for Raman analysis of surfaces in basis environment. Appl. Surf. Sci..

[cit19] Zhang J., Langille M. R., Personick M. L., Zhang K., Li S., Mirkin C. A. (2010). Concave Cubic Gold Nanocrystals with High-Index Facets. J. Am. Chem. Soc..

[cit20] Xue C., Chen X., Hurst S. J., Mirkin C. A. (2007). Self-assembled monolayer mediated silica coating of silver triangular nanoprisms. Adv. Mater..

[cit21] Dovgolevsky E., Haick H. (2008). Direct observation of the transition point between Quasi-spherical and cubic nanoparticles in a two-step seed-mediated growth method. Small.

[cit22] Kudelski A. (2009). Surface-enhanced Raman scattering study of monolayers formed from mixtures of 4-mercaptobenzoic acid and various aromatic mercapto-derivative bases. J. Raman Spectrosc..

[cit23] Taniguchi I., Yoshimoto S., Yoshida M., Kobayashi S., Miyawaki T., Aono Y., Sunatsuki Y., Taira H. (2000). Simple methods for preparation of a well-defined 4-pyridinethiol modified surface on Au(111) electrodes for cytochrome c electrochemistry. Electrochim. Acta.

[cit24] Aslan K., Wu M., Lakowicz J. R., Geddes C. D. (2007). Fluorescent core-shell Ag@SiO_2_ nanocomposites for metal-enhanced fluorescence and single nanoparticle sensing platforms. J. Am. Chem. Soc..

[cit25] Lu Y., Yin Y., Li Z. Y., Xia Y. (2002). Synthesis and Self-Assembly of Au@SiO_2_ Core-Shell Colloids. Nano Lett..

[cit26] Hackler R. A., Large N., Masango S. S., McAnally M. O., Schatz G. C., Stair P. C., Van Duyne R. P., Henry A.-I. (2016). High-Resolution Distance Dependence Study of Surface-Enhanced Raman Scattering Enabled by Atomic Layer Deposition. Nano Lett..

